# Serum MicroRNA Signatures Identified by Solexa Sequencing Predict Sepsis Patients’ Mortality: A Prospective Observational Study

**DOI:** 10.1371/journal.pone.0038885

**Published:** 2012-06-15

**Authors:** Huijuan Wang, Pengjun Zhang, Weijun Chen, Dan Feng, Yanhong Jia, Lixin Xie

**Affiliations:** 1 Department of Respiratory Medicine, Hainan Branch of Chinese PLA General Hospital, Sanya, Hainan Province, China; 2 Medical College, Nankai University, Tianjin, People’s Republic of China; 3 Department of Respiratory Medicine, Chinese PLA General Hospital, Beijing, China; 4 Department of Clinical Biochemistry, Chinese PLA General Hospital, Beijing, China; 5 Key Laboratory of Genome Sciences and Information, Beijing Institute of Genomics, Chinese Academy of Sciences, Airport Industry B6, Beijing, China; 6 Department of Medical Statistics, Chinese PLA General Hospital, Beijing, China; University of Massachusetts Medical School, United States of America

## Abstract

**Background:**

Sepsis is the leading cause of death in Intensive Care Units. Novel sepsis biomarkers and targets for treatment are needed to improve mortality from sepsis. MicroRNAs (miRNAs) have recently been used as finger prints for sepsis, and our goal in this prospective study was to investigate if serum miRNAs identified in genome-wide scans could predict sepsis mortality.

**Methodology/Principal Findings:**

We enrolled 214 sepsis patients (117 survivors and 97 non-survivors based on 28-day mortality). Solexa sequencing followed by quantitative reverse transcriptase polymerase chain reaction assays was used to test for differences in the levels of miRNAs between survivors and non-survivors. miR-223, miR-15a, miR-16, miR-122, miR-193*, and miR-483-5p were significantly differentially expressed. Receiver operating characteristic curves were generated and the areas under the curve (AUC) for these six miRNAs for predicting sepsis mortality ranged from 0.610 (95%CI: 0.523–0.697) to 0.790 (95%CI: 0.719–0.861). Logistic regression analysis showed that sepsis stage, Sequential Organ Failure Assessment scores, Acute Physiology and Chronic Health Evaluation II scores, miR-15a, miR-16, miR-193b*, and miR-483-5p were associated with death from sepsis. An analysis was done using these seven variables combined. The AUC for these combined variables’ predictive probability was 0.953 (95% CI: 0.923–0.983), which was much higher than the AUCs for Acute Physiology and Chronic Health Evaluation II scores (0.782; 95% CI: 0.712–0.851), Sequential Organ Failure Assessment scores (0.752; 95% CI: 0.672–0.832), and procalcitonin levels (0.689; 95% CI: 0.611–0.784). With a cut-off point of 0.550, the predictive value of the seven variables had a sensitivity of 88.5% and a specificity of 90.4%. Additionally, miR-193b* had the highest odds ratio for sepsis mortality of 9.23 (95% CI: 1.20–71.16).

**Conclusion/Significance:**

Six serum miRNA’s were identified as prognostic predictors for sepsis patients.

**Trial Registration:**

ClinicalTrials.gov NCT01207531

## Introduction

Sepsis is a state of sustained infection that results in a severe systemic inflammatory response and, possibly, shock [1]. Despite significant improvements in critical care medicine during recent decades, sepsis remains as one of the leading causes of mortality in the intensive care unit (ICU) [2]. Numerous studies have attempted to identify biomarkers to predict sepsis mortality, including serum C-reactive protein (CRP), Procalcitonin (PCT) levels [3], and Acute Physiology and Chronic Health Evaluation II scores (APACHE II) and Sequential Organ Failure Assessment scores (SOFA) [4].

However, CRP levels on the day of sepsis diagnosis have proved to have poor predictive value for mortality [5]. PCT, APACHE II scores, and SOFA scores have shown better predictive values compared to CRP levels; however, their areas under receiver operating characteristic (AUROC) curves were only 0.75 (95% CI: 0.66–0.85) [6], 0.75 (95% CI: 0.67–0.83), and 0.8 (95% CI: 0.72–0.88), respectively [7]. Thus, the sensitivities and specificities of these four biomarkers, which are widely used in clinical practice, are not sufficiently high to predict sepsis mortality.

MicroRNAs (miRNAs) are endogenous RNAs of ∼22 nt that play important regulatory roles by targeting mRNAs for cleavage or translational repression [8]. Recently, serum or plasma miRNAs levels have been shown to be stable and reproducible [9], [10], and the unique expression patterns of serum miRNAs can be used as finger prints for evaluating the prognoses of various diseases [9], [11]. To our knowledge, only miR-223 and miR-146a have shown diagnostic value for sepsis and only one plasma miRNA, miR-150, was identified as a potential biomarker for sepsis prognosis [12]. As is well known, sepsis is a complex syndrome that involves multiple organs and tissues, and not only leukocytes, and a single biomarker is not sufficient to reflect the severity of sepsis. Thus, a panel of biomarkers is needed to evaluate sepsis prognosis.

There have been no studies that used genome-wide screening of serum or plasma for sepsis prognosis or mortality from sepsis. Numerous methods have been used to detect the genome-wide expression profiles of miRNAs, such as Exqion miRCURY LNA array technology, the Illumina BeadChip platform, the Febit automated Geniom Real Time Analyzer platform, and Affymetrix GeneChip miRNA array technology [13]–[15]. However, all of these microarrays are limited due to the possibility of false positive results. Next generation sequencing is now being used to detect miRNA expression profiles in the sera of patients with different diseases [16], [17]. Compared to microarrays, these have shown better detection capabilities both in terms of the quality and quantity of miRNAs. These results showed that next generation sequencing is a promising method for genome-wide miRNA screening [18].

In this study, we first used Solexa sequencing to screen for serum miRNAs among 9 surviving and 9 non-surviving sepsis patients. Then, qRT-PCR was used to validate the Solexa sequencing results in a cohort of 166 sepsis patients. After validation, the predictive values of the screened miRNAs were compared to those of currently used clinical biomarkers. A multivariable logistic regression analysis was also used to evaluate the predictive values and odds ratios of these miRNAs.

## Results

### Clinical Characterizations of the Sepsis Patients

From July 2010 to March 2011, a total of 214 sepsis patients were screened. Based on 28-day mortality, they included 117 survivors and 97 non-survivors who were matched by sex (p = 0.406) and age (p = 0.730). Pulmonary infection was the leading cause of sepsis in these patients. There were no significant differences in the causes of sepsis between the two groups (pulmonary infection, p = 0.179; post-surgery, p = 0.591; acute pancreatitis, p = 0.981; multiple trauma, p = 0.491). However, APACHE II scores and SOFA scores were significantly different between sepsis survivors and non-survivors (**Table 1**).

**Table 1:** Clinical data for 214 sepsis patients.

Variables	Survivors (n = 117)	Non-survivors (n = 97)	P-value
Male/female	72/36	58/37	0.406^A^
Ages (years)	60.7±18.9	59.6±18.8	0.730^B^
Cause of sepsis (%)			
Pulmonary infection	39(33.3%)	41(42.3%)	0.179^A^
Post-surgery	30(25.6%)	23(23.7%)	0.591^A^
Acute pancreatitis	10(8.5%)	7(7.2%)	0.981^A^
Multiple trauma	17(14.6%)	11(11.3%)	0.491^A^
Others	21(18.0%)	15(15.5%)	0.629^A^
Intercurrent conditions			
CHD^#^	15(12.8%)	10(10.3%)	0.569^A^
Cancer	9(7.7%)	13(13.4%)	0.171^A^
Hypertension	21(17.9%)	17(17.5)	0.936^A^
Diabetes	7(6.0%)	5(5.2%)	0.793^A^
COPD*	25(21.4%)	20(17.1%)	0.894^A^
SOFA score	5.80±3.33	9.54±3.33	<0.001^B^
APACHE II score	15.75±6.46	22.72±6.62	<0.001^B^
CRP (mg/dl)	3.5(0.2,6.2)	12.15(6.7,32.8)	0.687^C^
PCT (ng/ml)	1.97(0.05,3.78)	8.29(3.91,191.44)	<0.001^C^

A: Chi Square tests; B: Student t test; C: Mann-Whitney U tests; #: Coronary heart disease;

* : Chronic obstructive pulmonary disease.

### Genome-wide Screening for Serum miRNAs by Solexa Sequencing

We selected 9 survivors and 9 non-survivors who were matched by sex, age, and sepsis severity for initial Solexa sequencing (**Table 2**). This sequencing detected 153 known miRNAs in the non-surviving group and 173 known miRNAs in the surviving group. The expression profiles of these miRNAs are shown in **Table S1 and Table S2**. Then, we compared the expressions of these known miRNA between the two groups; a large number of miRNAs were differentially expressed (Table S3).

**Table 2:** Clinical characteristics of the sepsis patients used for Solexa sequencing.

Variables	Survivors(n = 9)	Non-survivors(n = 9)	p-value
Male/female	6/3	7/2	0.599^A^
Ages (years)	62.3±20.8	60.9±21.5	0.887^B^
Type			
Sepsis	3	3	-
Severe sepsis	3	3	-
Septic shock	3	3	-
SOFA scores	6.0±3.7	9.6±3.6	0.057^B^
APACHE II	14.5±6.5	20.9±6.0	0.060^B^
CRP (mg/dl)	13.8 (6.1, 20.6)	14.15 (0.5, 22.2)	0.116^B^
PCT (ng/ml)	4.87(0.37,10.36)	10.18(0.45,32.76)	0.404^B^
WBC (×10^9^/L)	11.84(3.01,23.29)	10.08(7.62,29.6)	0.559^B^

A: Chi Square tests; B: Mann-Whitney U tests.

The level of an miRNA was considered to be significantly differentially expressed only if two criteria were both satisfied: (1) there were at least 20 copies in either the surviving or non-surviving group [16]; and (2) there was at least a two-fold different expression level between the two groups [16], [19]. After screening, 11 miRNAs were identified. These included 6 up-regulated miRNAs (miR-378, miR-483-5p, miR-193b*, miR-122, miR-499-5p, miR-206) and 5 down-regulated miRNAs (miR-451, miR-16, miR-15b, miR-15a, miR-486-5p) in the non-surviving group compared to the surviving group; these are shown in **Table 3**. Despite its low fold-change of 1.8, miR-223 was also chosen because miR-223 had been previously proven to be a biomarker of sepsis [20]. Thus, 12 miRNAs were selected for a small sample size validation by qRT-PCR.

**Table 3:** Fold-changes of selected miRNAs in Non-survivors (D) and Survivors (S).

miR-names	Fold-change*	p-value
Down-regulated innon-survivors’ sera		
hsa-miR-451	–3.27	0
hsa-miR-16	–3.21	0
hsa-miR-15b	–3.07	0
hsa-miR-15a	–2.28	<0.001
hsa-miR-486-5p	–2.19	0
hsa-miR-223	–1.81	<0.001
Up-regulated innon-survivors’ sera		
hsa-miR-378	2.38	<0.001
hsa-miR-483-5p	2.05	0
hsa-miR-193b*	2.77	0
hsa-miR-122	3.14	0
hsa-miR-499-5p	4.40	0
hsa-miR-206	4.88	0

* Fold change formula: Fold-change = log2 (D/S).

### qRT-PCR Small Sample Size Validation and Large Sample Size Confirmation

24 healthy controls were used to normalize the expression levels of the miRNAs measured in our study and all of these miRNAs were detectable in normal controls’ sera. Based on previous results [21], [22], U6 snRNA was selected as an internal normalization control for miRNA qPCR. In our study, there were no significant differences in raw Ct values for U6 snRNA among normal controls, non-survivors, and survivors (p = 0.376; Kruskal-Wallis H).

**Figure 1:**
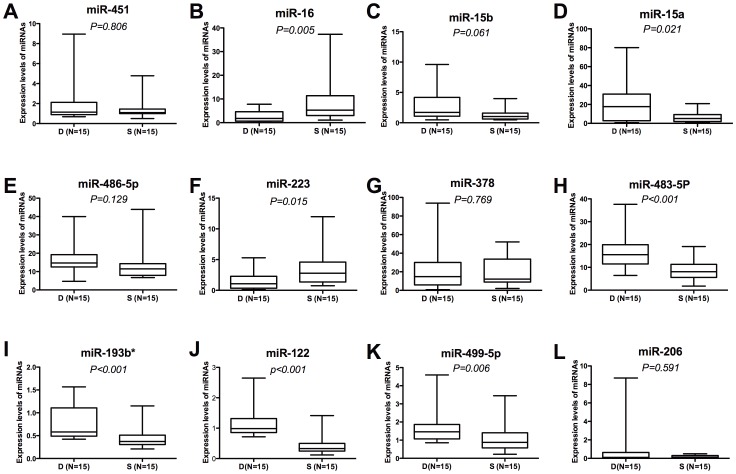
Comparison of 12 miRNAs expression levels between sepsis non-survivors and survivors in a validation set. Differentially expressed miRNAs identified by Solexa sequencing were validated by qRT-PCR in sepsis survivors (S; N = 15) compared to non-survivors (D; N = 15). MiR-16 (*p = 0.005*), miR-15a (*p = 0.021*), miR-223 (*p = 0.015*), miR-483-5p (*p<0.001*), miR-193b* (*p<0.001*), miR-122 (*p<0.001*), and miR-499-5p (*p = 0.006*) were significantly different after validation with qRT-PCR. Expression levels of the 12 miRNAs were normalized to U6 snRNA above normal controls and given as fold-changes (2^–ΔΔCt^ ). △△Ct  =  (Ct_miRNA_-Ct_U6__snRNA_) _patients_-(Ct_miRNA_-Ct_U6 snRNA_)_controls_. Mann-Whitney U-test was used for statistical comparisons.

We used qRT-PCR to validate the altered expressions of the 12 candidate miRNAs for 15 survivors and 15 non-survivors. After validation, seven miRNAs were identified (associated p-values in parentheses): miR-223 (p = 0.015); miR-15a (p = 0.005); miR-16 (p = 0.021); miR-499-5p (p = 0.006); miR-122 (p<0.001), miR-193b*(p<0.001) and miR-483-5p (p<0.001). Compared to the surviving group, miR-16 and miR-223 were up-regulated in the non-surviving group and the levels of miR-15a, miR-499-5p, miR-193b*, miR-122, and miR-483-5p were up-regulated (**Figure 1**). Although levels of miR-15b were not significantly different in the validation set, previous study has proved that levels of miR-15b were significantly higher than other expressed miRNAs in T cell subsets [23]. Hence, miR-15b was also selected for further confirmation.

**Figure 2:**
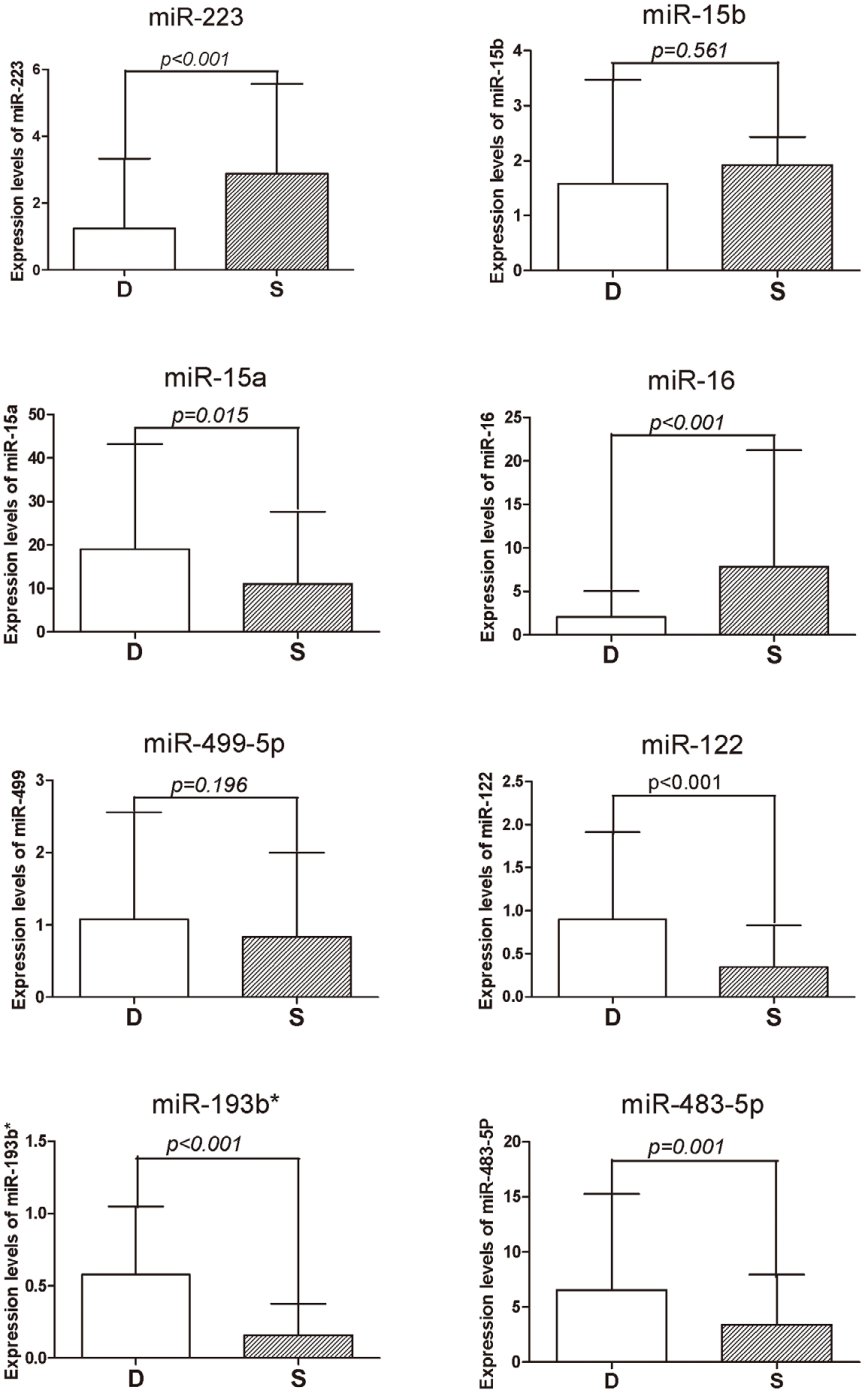
Expression levels of 8 miRNAs in sepsis non-survivors and survivors in a confirmation set. These 8 miRNAs were significantly differentially expressed between sepsis non-survivors and survivors after qRT-PCR validation in a smaller study sample. Then, these were checked by qRT-PCR in a larger study sample size (Non-survivors = D, n = 73; Survivors = S, n = 93). Only 6 of the 8 miRNAs remained as significantly different between the D group and S groups. Expression levels of these 8 miRNAs were normalized to U6 snRNA U6 snRNA above normal controls and given as fold-changes (2^–ΔΔCt^ ), △△Ct =  (Ct_miRNA_-Ct_U6__snRNA_)_patients_-(Ct_miRNA_-Ct_U6 snRNA_)_controls_. Mann-Whitney U-test or student t-test was used for statistical comparisons.

Next, the eight differentially expressed miRNAs were further validated in a confirmation set comprised of 93 survivors and 73 non-survivors. As a result, two of the eight miRNAs’ levels (miR-15b and miR-499-5p) were not significantly different. Six miRNAs were confirmed to be significantly differentially expressed and these miRNA expression pattern alterations in the confirmation set were consistent with those of the validation set. The expression levels of miR-15a (p = 0.015), miR-193b* (p<0.001), miR-122 (p<0.001), and miR-483-5p (p<0.001) in non-survivors were significantly higher than in survivors, and the levels of miR-223(p<0.001) and miR-16 (p<0.001) were significantly lower (**Figure 2**).

### Comparisons of the Predictive Mortality Values of the Six miRNAs with SOFA Scores, APACHE II Scores, and PCT Levels

**Figure 3:**
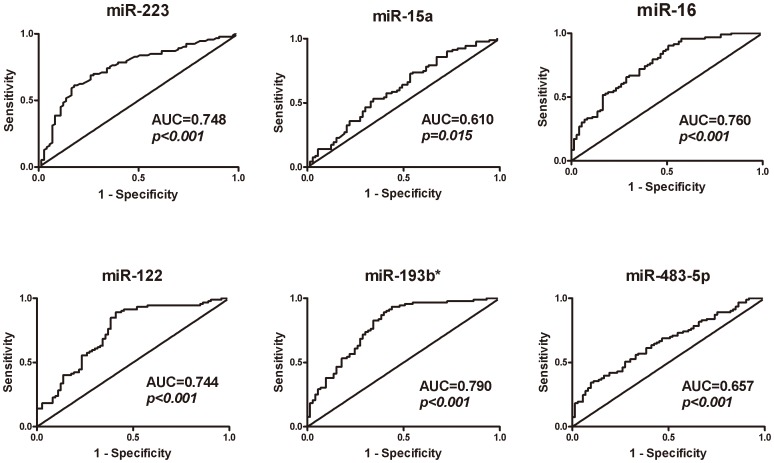
Receiver operating characteristic (ROC) curves for serum miRNAs for sepsis non-survivors (n = 73) and survivors (n = 93). These six miRNAs were finally confirmed by qRT-PCR. Areas under the ROC curves are also shown.

**Figure 4:**
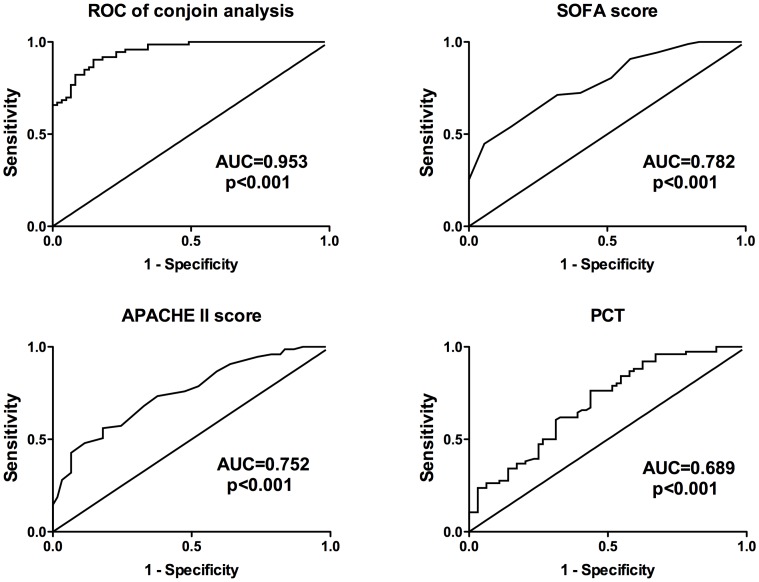
Receiver operating characteristic (ROC) curves for serum miRNAs and clinically used indicators for sepsis non-survivors (n = 73) and survivors (n = 93). Serum levels of miRNAs were quantified using real time qPCR. Each qPCR was done in triplicate in 96-well plates. Expression levels of the selected miRNAs were normalized to U6 snRNA and presented as fold-changes (2^-ΔΔCt^) above normal controls.

To investigate the predictive mortality value of the six confirmed miRNAs, receiver operating characteristic (ROC) curves were generated and the areas under the curves (AUCs) were calculated. The AUCs for the six miRNAs ranged from 0.610 (95%CI: 0.523–0.697) to 0.790 (95%CI: 0.719–0.861) **(** **Figure 3** **)**. The AUCs for SOFA scores, APACHE II scores, and PCT levels were, respectively, 0.782 (95%CI: 0.712–0.851), 0.752 (95%CI: 0.672–0.832), and 0.689 (95%CI: 0.611–0.784) (**Figure 4**). The AUC for miR-193b* had the highest value, followed by those for SOFA scores and miR-16. AUCs for miR-223 and miR-122 were nearly identical to the AUC for APACHE II scores.

### Four Serum miRNAs Identified as Risk Factors for Sepsis Patients’ Prognosis

Logistic regression analysis was used to evaluate possible associations between the six miRNAs and sepsis patients’ mortality. First, univariate logistic regression was used for the six miRNAs along with sepsis stage (sepsis = 1, severe sepsis = 2, septic shock = 3), age, sex, SOFA scores, APACHE II scores, CRP and PCT levels, white blood cell counts (WBC), coronary heart diseases (CHD), diabetes, hypotension, and COPD. The sepsis stage, six serum miRNAs, SOFA scores, APACHE II scores, PCT levels and diabetes were significantly different between survivors and non-survivors (all p<0.05). These variables were used in multivariable logistic regression analysis.

Only the sepsis stage, SOFA scores, APACHE II scores, miR-15a, miR-16, miR193b*, and miR-483-5p were entered into the final regression equation (**Table 4**). The predictive values of these seven variables were evaluated by ROC analysis. The AUC of these combined seven variables was 0.953 (95%CI: 0.923–0.985), which was much higher than SOFA scores, APACHE II scores, or any of the miRNAs (**Figure 4**). When the cut-off point was set at 0.550, the predictive value of the seven variables had a sensitivity of 88.5% and a specificity of 90.4%. Then, we calculated the predictive value when only SOFA and APACHE II scores were combined. The AUC was 0.891 (95%CI: 0.843–0.939), which was lower than when the miRNAs results were added.

**Table 4:** Multivariate logistic regression analysis of the variables (Forward conditional).

Variables	Univariate		Multivariate	
	ORs	p-value	ORs	p-value
Sepsis stage	3.15(1.94, 5.10)	<0.001	6.96(2.11,22.99)	0.001
Sex	1.51(0.78, 2.92)	0.23	-	-
Ages	0.99(0.98, 1.00)	0.73	-	-
SOFA score	1.37(1.23, 1.53)	<0.001	1.25(1.01, 1.54)	0.039
APACHE II	1.18(1.10, 1.26)	<0.001	1.14(1.02,1.27)	0.026
CRP	1.02(0.98, 1.07)	0.33	-	-
PCT	1.06(1.03, 1.10)	0.001	-	-
WBC	1.01(0.96, 1.06)	0.817	-	-
Serum miR-223	0.67(0.54, 0.82)	<0.001	-	-
Serum miR-15a	1.02(1.00, 1.04)	0.022	1.03(1.00, 1.07)	0.037
Serum miR-16	0.81(0.72, 0.91)	<0.001	0.60(0.45,0.81)	0.001
Serum miR-122	5.23(2.40,11.41)	0.02	-	-
Serum miR-193b*	37.88(10.64,134.90)	<0.001	9.07(1.32,62.42)	0.025
Serum miR-483-5p	1.08(1.01, 1.15)	0.007	1.29(1.08,1.55)	0.006
CHD	1.31(0.55, 3.11)	0.548	_	_
Diabetes	3.74(1.14, 12.31)	0.030	_	_
Hypotension	1,70(0.70, 4.14)	0.245	_	_
COPD	1.37(0.52, 3.58)	0.525	_	_

Among these seven factors, miR-193b* had the highest odds ratio (OR) of 9.07 (95% CI: 1.32–62.42), followed by sepsis stage, miR-483-5p, SOFA scores, APACHE II scores, miR-15a, and miR-16 with ORs, respectively, of 6.96 (95% CI: 2.11–22.99), 1.29 (95% CI: 1.08–1.55), 1.25 (95%CI: 1.01, 1.54), 1.14 (95% CI: 1.02–1.27), 1.03 (95% CI: 1.00–1.07), and 0.60 (95% CI: 0.45–0.81). These results indicated that sepsis stage, miR-193b*, miR-483-5p, APACHE II scores, and miR-15a were independent risk factors for sepsis patients’ death. miR-16 was identified as a factor associated with decreased risk of death for sepsis patients.

## Discussion

Several circulating miRNAs have recently been reported to be biomarkers for sepsis diagnosis and prognosis [12], [20]. In our study, a genome-wide Solexa method was first used to screen 18 sepsis patients’ sera for miRNAs. Then, qRT-PCR was used for 196 sepsis patients to confirm the results of Solexa sequencing. Two methods for validation and a large sample size made our results more convincing. As a result, six miRNAs were confirmed to be significantly differentially expressed between sepsis survivors and non-survivors.

Among these six miRNAs, the predictive value of miR-193b* for sepsis mortality was better than SOFA scores and APACHE II scores, which are both composites that integrate numerous sepsis indicators. However, serum miR-15a and miR-483-5p had poor predictive values for sepsis mortality. Thus, we used a logistic regression analysis to select those miRNAs that were associated with death from sepsis. These results showed that a combination of miR-15a, miR-16, miR-193b*, miR-483-5p, SOFA scores, APACHE II scores, and sepsis stage had a much better predictive value for mortality. Therefore, if we were to integrate these six miRNAs into a composite indicator in clinical practice, this composite indicator would have a much better predictive value for sepsis mortality than SOFA scores and APACHE II scores.

These six miRNAs were differentially expressed even when survivors and non-survivors were matched by sepsis severity. Hence, these biomarkers may be genetic markers and may not necessarily be suitable for characterizing sepsis progression. Functional studies involving these biomarkers will be needed, which may provide additional valuable information for treating physicians.

Previous studies of miRNAs in sepsis patients primarily focused on the miRNA expression profiles of WBCs. miR-146b, miR-150, miR-342, and miR-let-7 g were found to be differentially expressed by WBCs from healthy donors after treatment with *E. Coli* lipopolysaccharide (LPS) infusion for 4 hours [24], and miR-150, miR-182, miR-342-5p, and miR-486 were identified in WBCs from sepsis patients’ peripheral blood [12]. However, none of these miRNAs were present in the expression profiles of our six miRNAs. These differences may be due to the following reasons.

In peripheral blood serum, circulating miRNAs are found in microvesicles (MVs), which have been identified as carriers of genetic information exchange between cells [25]. The majority of peripheral blood MVs derive from platelets [26], [27] and a second, large population of MVs derives from mononuclear phagocyte cell lineages. Only a small percentage of MVs are derived from T-cells and neutrophils [27]. In sepsis patients, WBCs are not the only blood cells that are involved in sepsis pathogenesis. Thus, the serum miRNAs screened for using a genome-wide method might derive mostly from other sources given the small percentage of miRNAs from white cells.

Functional studies of these screened miRNAs in our study have already been performed by other investigators. miR-223, miR-15a, and miR-16 have been found to be associated with the innate immune system by targeting IKKα mRNA, which is involved in the NF-κB signaling pathway [28]. Functional studies of miR-193b* have mostly focused on cancer [29], [30]. miR-122, a liver-specific miRNA, was found to be associated with hepatocellular carcinoma [31] and chronic hepatitis [32]. Abnormal expression of miR-483-5p was indicative of a poor prognosis for adrenocortical carcinomas [33]. To date, no direct functional study has shown that these miRNAs were associated with sepsis. Hence, much work needs to be done.

This study was novel in several respects. First, we addressed a significant medical condition-sepsis-from a new perspective: the involvement of serum miRNAs. Second, Solexa sequencing was first used for genome-wide screening of sepsis patients’ sera to identify differentially expressed miRNAs. Third, this was the first time that a high- throughput method was used to screen for serum miRNAs to evaluate sepsis prognosis. Finally, this is the first report to show that miR-16, miR-15a, miR-193b*, and miR-483-5p were associated with sepsis prognosis.

However, this study had some limitations. We only used 9 survivors and 9 non-survivors in our discovery set. Although six miRNAs were found to be valuable for predicting mortality of sepsis patients, a number of miRNAs with low expression levels might have been left out during the initial screening by Solexa sequencing. The 9 samples in each group were comprised of 3 sepsis patients, 3 severe sepsis patients, and 3 septic shock patients. Solexa sequencing performed only for single subgroups may discover some specific miRNAs in these subgroups. Another limitation was that miR-499 were significantly differentially expressed in the validation set with p value of 0.006 but not in confirmation set with value of 0.196. This result meant that relatively small numbers in sample groups could readily give misleading results and these results of our study were still needed to be validated in a much larger sample group. In addition, conditions of the patients and comorbidity influences may also affect the results. In addition, in our study, we only evaluated the levels of these miRNAs in patients who were admitted to ICU within 24 hours. Whether there is a progression or increase of these biomarkers when these patients approach death is still unknown. Hence, evaluation of the dynamic changes of these miRNAs during the sepsis process was essential to our further study.

Regardless of these limitations, the six miRNAs were found to be valuable predictors of sepsis mortality. In future studies, a focus on the trends of the expression level changes of these six miRNAs during hospitalization in the ICU would be even more valuable. In addition, predicting the target genes of these six miRNAs and post-experimental validation of these target genes may provide new targets for the treatment of sepsis.

In conclusion, six serum miRNAs identified in our study can be used to predict sepsis patients’ mortality.

## Materials and Methods

The protocol for this trial and supporting CONSORT checklist are available as supporting information; see Checklist S1 and Protocol S1.

### Ethics

Written informed consent was obtained from all sepsis patients and normal controls. This study was approved by the Committee on Ethics of the Chinese PLA General Hospital.

### Study Subjects

All patients who were diagnosed with sepsis met the definitions of the American College of Chest Physicians/Society of Critical Care Medicine (ACCP/SCCM) [34]. Patients younger than 18 years of age or those who were immunosuppressed were excluded from this study. Patients who did not receive adequate treatment because of economic hardship were also excluded. A total of 214 sepsis patients (117 survivors and 97 non-survivors) were enrolled from July 2010 to March 2011. Healthy controls (n = 24) were recruited from the Health Screening Center of the Chinese PLA General Hospital and they did not have any type of known infection or known medical condition.

Based on 28-day mortality, we divided sepsis patients into a surviving group and a non-surviving group. First, to detect differently expressed miRNAs between the surviving and non-surviving groups, 9 survivors and 9 non-survivors who were matched by sex (p = 0.902), age (p = 0.887), and sepsis severity (SOFA scores, p = 0.057; APACHE II scores, p = 0.060) were screened by Solexa sequencing (miRBase 15.0). Next, qRT-PCR was used in a validation set (15 survivors and 15 non-survivors) who were also matched by sex (p = 0.821), age (p = 0.329), and sepsis severity (SOFA scores, p = 0.089; APACHE II scores, p = 0.071) to validate the Solexa sequencing results in a small sample size. Finally, the validated serum miRNAs in the small sample size were confirmed in a confirmation set (93 survivors and 73 non-survivors).

### Blood Samples and Serum Total RNA Isolation

Blood samples of all the patients were drawn within 24 hours after a diagnosis of sepsis was made. Coagulated blood samples were collected in tubes containing a separating gel and clot activator and centrifuged at 3000 rpm for 15 min at room temperature. The supernatant was transferred to Eppendorf tubes. These samples were centrifuged at 15,000 rpm for 30 min to precipitate cell debris, and the supernatants were stored at -80°C until RNA extraction. Serum total RNA was isolated using a mirVana PARIS kit (Ambion, #1556) according to the manufacturer ‘s instructions. All serum RNA preparations were pre-treated with RNase-free DNase I (Promega) to eliminate potential DNA contamination.

### Solexa Sequencing and Bioinformatics Analysis

The serum samples of 9 survivors and 9 non-survivors were pooled for their respective groups. Total RNA was purified directly for Solexa sequencing analysis using an Illumina Genome Analyzer (Illumina, San Diego, CA, USA) according to the manufacturer's instructions [35]. Briefly, a pair of Solexa adaptors were ligated to the 5' and 3' ends of total RNA, and small RNA molecules were amplified by reverse transcription PCR (RT-PCR) using a RT-PCR kit (Invitrogen). The adaptor primers for 17 cycles and fragments around 30 bp (small RNA + adaptors) were isolated from an agarose gel. Purified RNA was used directly for cluster generation and sequencing analysis using an Illumina Genome Analyzer (Illumina, San Diego, CA, USA) according to the manufacturer's instructions. After a bioinformatics analysis [36], the remaining miRNAs were searched against the Sanger miRBase (version15.0) [37] to identify conserved, known miRNAs. Then, the fold-changes of the known miRNAs between the two groups were determined by: Log_2_(copies of non-survivors/copies of survivors). This work was done at the Beijing Genomics Institute (BGI).

### qRT-PCR Validation

After total RNA isolation, reverse transcription (RT) was done in a reaction volume of 7.5 µL, which contained 2.08 µL of water, 0.75 µL of 10× reverse transcription buffer, 0.095 µL of RNase inhibitor, 0.075 µL of dNTPs, 0.5 µL of Multiscribe reverse transcriptase, 1.5 µL of miRNA-specific stem-loop RT primer (Applied Biosystems), and 2.5 µL (5 ng) of digested RNA preparations. RT used a Mastercycler Ep Gradient at 16°C for 30 min, 42°C for 30 min, and 85°C for 5 min. Real-time qPCR used a TaqMan MicroRNA assay (Applied Biosystems). Briefly, 4.5 µL of 5∶28 diluted RT product was combined with 5.0 µL of TaqMan gene expression Master Mix and 0.5 µL of Taqman miRNA assay. qPCR used an ABI PRISM 7300 detection system at 95°C for 5 min, followed by 40 cycles of 95°C for 15 s and 60°C for 1 min. All qPCR reactions were performed in triplicate and the raw Ct (threshold cycle) of each sample was the mean value of three Ct values. The data were analyzed by the 2^-ΔΔCT^ method.

### CRP and PCT Determinations

Serum CRP levels were determined using scattering turbidimetry (CardioPhase hsCRP, Siemens, Germany) and PCT was determined using an enzyme-linked fluorescence analysis kit (ELFA, VIDAS BRAHMS PCT kit, bioMerieux SA, France). All assays were done according to the manufacturer’s instructions and duplicate measurements were made.

### Statistical Methods

Expression levels of selected miRNAs detected by qRT-PCR were normalized to U6 snRNA and presented as the fold-change (2^-ΔΔCt^) above the normal control: △△Ct  =  (Ct_miRNA_-Ct_U6__snRNA_)_patients_-(Ct_miRNA_-Ct_U6 snRNA_)_controls._ Results for normally distributed continuous variables are given as means (±standard errors of the mean) and compared between groups by Student’s t-tests. Results for non-normally distributed continuous variables are summarized as medians (interquartile ranges) and were compared by Mann-Whitney U tests. To evaluate the predictive values of selected miRNAs, ROC curves were generated and the areas under the curves were determined. Multivariable logistic regression analysis was used to screen for independent risk or protective factors for sepsis. Then, an analysis of a combination of these screened variables was done and areas under the curve for the predictive probabilities of these variables were also determined. *P*-values <0.05 were considered statistically significant. All statistical analyses used SPSS software (version 16.0).

## Supporting Information

Checklist S1 **CONSORT Checklist.**
(DOC)

Protocol S1 **Trial Protocol.**
(PDF)

Table S1 **Expression profiles of serum miRNAs in non-surviving sepsis patients (n = 9) detected by Solexa sequencing.**
(DOC)

Table S2 **Expression profiles of serum miRNAs in surviving sepsis patients (n = 9) detected by Solexa sequencing.**
(DOC)

Table S3 **Comparisons of miRNAs between 9 survivors (S) and 9 non-survivors (D) detected by Solexa sequencing.**
(XLS)
