# Supplementary material for: Serum MicroRNA Signatures Identified by Solexa Sequencing Predict Sepsis Patients’ Mortality: A Prospective Observational Study
Source: PLoS One. 2012 Jun 15;7(6):e38885. doi: 10.1371/journal.pone.0038885 (PMC3376145; doi:10.1371/journal.pone.0038885)
Supplement: Protocol S1 — Trial Protocol. (PDF) [file pone.0038885.s002.pdf]

Protocol Registration Receipt
09/22/2010

MiRNAs Evaluate the Prognosis of Sepsis (METPS)

This study is currently recruiting participants.
Verified by Chinese PLA General Hospital, September 2010

Sponsor:	Chinese PLA General Hospital
Collaborators:	Chinese PLA General Hospital
Information provided by:	Chinese PLA General Hospital
ClinicalTrials.gov Identifier:	NCT01207531

Purpose

Sepsis is a common cause of death in intensive care unit, timely and accurate diagnosis and treatment directly affect the survival rate. MiRNA is a post-transcriptional small RNA which regulate mRNA expression. The present study was designed to screen several miRNA by microarray which evaluate the sepsis prognosis in order to be a new target for the treatment of sepsis.

Condition
Sepsis

Study Type: Observational
Study Design: Case-Control, Prospective
Official Title: miRNA in the Evaluation of the Value of Sepsis Prognosis Prospective Observational Study

Further study details as provided by Chinese PLA General Hospital:

Biospecimen Retention: Samples Without DNA
human serum

Primary Outcome Measure:

- all cause mortality [Time Frame: 28days after admited in ICU] [Designated as safety issue: No]

Estimated Enrollment: 100
Study Start Date: July 2010
Estimated Study Completion Date: April 2011
Estimated Primary Completion Date: November 2010

Groups/Cohorts	Interventions
Survival Group	
Death group	

The study is a non-intervention, prospective observational study. Purpose of this sudy is to screening several miRNAs by microarray which can evaluate the prognosis of sepsis . We will collect serum samples from patients with sepsis in SICU, RICU and EICU of 301 Hospital since September 2009 , and then use the chip and qRT-PCR to Screen miRNAs which can evaluate the prognosis of sepsis, and statistical analysis the miRNAS expression correlation with SOFA score.

Eligibility

within 24 hours after admited in ICU

Sampling Method: Non-Probability Sample
Ages Eligible for Study: 18 Years and older
Genders Eligible for Study: Both
Accepts healthy volunteers.

Inclusion Criteria:

- Clinical diagnosis of sepsis
- Patients who agree with the study

Exclusion Criteria:

- Aged <18 years;
- Into the group who died within 24 hours;
- Agranulocytosis (<0.5 × 109 / L);
- Combined HIV infection.

Contacts and Locations

Contacts

Huijuan Wang, master +86 13466791738 wanghuijuan301@gmail.com

Locations

China, Beijing

Chinese PLA General Hospital Recruiting
Beijing, Beijing, China, 100853

Investigators

Study Director:	Lixin Xie, Doctor	Pneumology Department of chinese PLA General Hospital

More Information

Responsible Party: Pneumology Department of Chinese PLA General Hospital (Lixin Xie)
Study ID Numbers: 301PLAGH-2010915
Health Authority: China: Ethics Committee
