# Supplementary material for: Serum MicroRNA Signatures Identified by Solexa Sequencing Predict Sepsis Patients’ Mortality: A Prospective Observational Study
Source: PLoS One. 2012 Jun 15;7(6):e38885. doi: 10.1371/journal.pone.0038885 (PMC3376145; doi:10.1371/journal.pone.0038885)
Supplement: Table S1 — Expression profiles of serum miRNAs in non-surviving sepsis patients (n = 9) detected by Solexa sequencing. (DOC) [file pone.0038885.s003.doc]

Table S1. The expression profiles of serum miRNAs in non-survivors of sepsis patients (n=9) detected by Solexa sequencing.

| miRNAs | Death Group(copies) | miRNAs | Death Group(copies) |
| --- | --- | --- | --- |
| hsa-miR-122 | 787137 | hsa-miR-335 | 212 |
| hsa-miR-423-5p | 435402 | hsa-miR-1323 | 201 |
| hsa-miR-320a | 53216 | hsa-miR-184 | 191 |
| hsa-miR-140-3p | 45313 | hsa-miR-125a-5p | 184 |
| hsa-let-7f | 44358 | hsa-miR-200a | 178 |
| hsa-let-7a | 43548 | hsa-miR-30c-1* | 176 |
| hsa-let-7b | 35723 | hsa-miR-194 | 175 |
| hsa-miR-1 | 26801 | hsa-miR-3154 | 175 |
| hsa-miR-320b | 13759 | hsa-miR-127-3p | 170 |
| hsa-miR-185 | 12899 | hsa-miR-23b | 164 |
| hsa-miR-192 | 10069 | hsa-miR-151-5p | 162 |
| hsa-miR-101 | 9704 | hsa-miR-17 | 156 |
| hsa-miR-206 | 8623 | hsa-miR-30a* | 152 |
| hsa-miR-378c | 7573 | hsa-miR-139-3p | 148 |
| hsa-let-7c | 6234 | hsa-miR-92a-1* | 148 |
| hsa-miR-199a-3p | 4373 | hsa-miR-7 | 138 |
| hsa-miR-199b-3p | 4373 | hsa-miR-130a | 126 |
| hsa-miR-499-5p | 4065 | hsa-miR-93 | 116 |
| hsa-let-7i | 3888 | hsa-miR-106b | 113 |
| hsa-miR-21 | 3622 | hsa-miR-125b | 109 |
| hsa-miR-193b* | 3484 | hsa-miR-133a | 107 |
| hsa-miR-103 | 3426 | hsa-miR-144 | 105 |
| hsa-miR-483-5p | 3256 | hsa-miR-2110 | 100 |
| hsa-let-7g | 3253 | hsa-miR-130b | 99 |
| hsa-let-7d | 3101 | hsa-miR-363 | 99 |
| hsa-miR-486-5p | 2823 | hsa-miR-30e* | 93 |
| hsa-miR-92a | 2750 | hsa-miR-98 | 93 |
| hsa-miR-107 | 2604 | hsa-miR-500a* | 91 |
| hsa-miR-320c | 2071 | hsa-miR-765 | 91 |
| hsa-miR-1246 | 1966 | hsa-miR-485-5p | 91 |
| hsa-miR-22 | 1847 | hsa-miR-502-3p | 91 |
| hsa-let-7e | 1625 | hsa-miR-30e | 90 |
| hsa-miR-142-5p | 1620 | hsa-miR-877 | 85 |
| hsa-miR-16 | 1597 | hsa-miR-152 | 84 |
| hsa-miR-10a | 1515 | hsa-miR-26a | 78 |
| hsa-miR-221 | 1055 | hsa-miR-151-3p | 78 |
| hsa-miR-191 | 860 | hsa-miR-574-3p | 73 |
| hsa-miR-378 | 858 | hsa-miR-30c-2* | 73 |
| hsa-miR-1228* | 758 | hsa-miR-548l | 70 |
| hsa-miR-30a | 670 | hsa-miR-3190 | 68 |
| hsa-miR-29a | 663 | hsa-let-7d* | 68 |
| hsa-miR-30d | 652 | hsa-miR-149* | 68 |
| hsa-miR-320d | 618 | hsa-miR-31 | 67 |
| hsa-miR-26b | 589 | hsa-miR-1270 | 64 |
| hsa-miR-181a | 581 | hsa-miR-96 | 63 |
| hsa-miR-222 | 574 | hsa-miR-223* | 60 |
| hsa-miR-24 | 540 | hsa-miR-20b* | 49 |
| hsa-miR-223 | 532 | hsa-miR-424 | 48 |
| hsa-miR-15a | 504 | hsa-miR-186 | 47 |
| hsa-miR-148a | 472 | hsa-miR-183 | 47 |
| hsa-miR-302a* | 457 | hsa-miR-25 | 46 |
| hsa-miR-451 | 430 | hsa-miR-1307 | 44 |
| hsa-miR-10b | 422 | hsa-miR-210 | 40 |
| hsa-miR-92b* | 407 | hsa-miR-106b* | 37 |
| hsa-miR-27a | 390 | hsa-miR-3615 | 33 |
| hsa-miR-27b | 390 | hsa-miR-885-3p | 24 |
| hsa-miR-143 | 389 | hsa-miR-124 | 7 |
| hsa-miR-889 | 384 | hsa-miR-302a | 5 |
| hsa-miR-23a | 381 | hsa-miR-423-3p | 5 |
| hsa-miR-664* | 362 | hsa-miR-302d | 4 |
| hsa-miR-193a-5p | 355 | hsa-miR-323-3p | 4 |
| hsa-miR-92b | 319 | hsa-miR-302c* | 3 |
| hsa-miR-375 | 313 | hsa-miR-302c | 3 |
| hsa-miR-208b | 313 | hsa-miR-516b | 3 |
| hsa-miR-25* | 309 | hsa-miR-372 | 3 |
| hsa-miR-371-5p | 301 | hsa-miR-330-3p | 2 |
| hsa-miR-15b | 282 | hsa-miR-342-3p | 2 |
| hsa-miR-199b-5p | 268 | hsa-miR-432 | 2 |
| hsa-miR-23b* | 268 | hsa-miR-34c-5p | 2 |
| hsa-miR-302b | 267 | hsa-miR-187 | 2 |
| hsa-miR-744 | 266 | hsa-miR-9 | 2 |
| hsa-miR-503 | 259 | hsa-miR-411 | 2 |
| hsa-miR-99b | 245 | hsa-miR-200c | 2 |
| hsa-miR-99a | 238 | hsa-miR-940 | 2 |
| hsa-miR-128 | 227 | hsa-miR-182 | 2 |
| hsa-miR-215 | 220 | hsa-miR-340 | 2 |
| hsa-miR-181b | 213 |  |  |
