# Supplementary material for: Serum MicroRNA Signatures Identified by Solexa Sequencing Predict Sepsis Patients’ Mortality: A Prospective Observational Study
Source: PLoS One. 2012 Jun 15;7(6):e38885. doi: 10.1371/journal.pone.0038885 (PMC3376145; doi:10.1371/journal.pone.0038885)
Supplement: Table S2 — Expression profiles of serum miRNAs in surviving sepsis patients (n = 9) detected by Solexa sequencing. (DOC) [file pone.0038885.s004.doc]

Table S2. The expression profiles of serum miRNAs in survivors of sepsis patients (n=9) detected by Solexa sequencing.

| miRNAs | Copies | miRNAs | Copies |
| --- | --- | --- | --- |
| hsa-miR-423-5p | 480224 | hsa-miR-363 | 148 |
| hsa-miR-140-3p | 125404 | hsa-miR-130b | 147 |
| hsa-miR-122 | 97575 | hsa-miR-2110 | 146 |
| hsa-miR-320a | 62018 | hsa-miR-146b-5p | 146 |
| hsa-let-7f | 57105 | hsa-miR-342-3p | 143 |
| hsa-let-7b | 47896 | hsa-miR-411 | 137 |
| hsa-let-7a | 46509 | hsa-miR-371-5p | 135 |
| hsa-miR-185 | 23370 | hsa-miR-23b* | 134 |
| hsa-miR-16 | 16167 | hsa-miR-532-5p | 127 |
| hsa-miR-320b | 15505 | hsa-miR-31 | 119 |
| hsa-miR-486-5p | 14106 | hsa-miR-34c-5p | 118 |
| hsa-miR-1 | 12485 | hsa-miR-486-3p | 114 |
| hsa-miR-101 | 12459 | hsa-miR-339-3p | 113 |
| hsa-miR-192 | 7620 | hsa-miR-1298 | 112 |
| hsa-let-7c | 7358 | hsa-miR-193a-5p | 110 |
| hsa-let-7d | 6032 | hsa-miR-20a | 109 |
| hsa-miR-199a-3p | 5620 | hsa-miR-124 | 107 |
| hsa-miR-199b-3p | 5620 | hsa-miR-340 | 104 |
| hsa-miR-21 | 4788 | hsa-miR-30e* | 99 |
| hsa-miR-92a | 4566 | hsa-miR-627 | 97 |
| hsa-miR-451 | 4541 | hsa-miR-29c | 94 |
| hsa-miR-103 | 4078 | hsa-miR-485-5p | 93 |
| hsa-let-7g | 3684 | hsa-miR-424* | 91 |
| hsa-miR-107 | 3286 | hsa-miR-524-5p | 88 |
| hsa-miR-142-5p | 2725 | hsa-miR-375 | 83 |
| hsa-miR-15a | 2675 | hsa-miR-130a | 82 |
| hsa-miR-15b | 2604 | hsa-miR-122* | 82 |
| hsa-let-7i | 2568 | hsa-miR-23a* | 80 |
| hsa-miR-378c | 2125 | hsa-miR-125b | 74 |
| hsa-miR-223 | 2050 | hsa-miR-30a* | 73 |
| hsa-miR-320c | 1875 | hsa-miR-29b | 73 |
| hsa-let-7e | 1735 | hsa-miR-133a | 72 |
| hsa-miR-191 | 1535 | hsa-miR-96 | 72 |
| hsa-miR-1228* | 1433 | hsa-miR-134 | 71 |
| hsa-miR-221 | 1298 | hsa-miR-664* | 69 |
| hsa-miR-29a | 934 | hsa-miR-1307 | 68 |
| hsa-miR-483-5p | 860 | hsa-miR-150 | 68 |
| hsa-miR-22 | 823 | hsa-miR-425 | 68 |
| hsa-miR-25* | 745 | hsa-miR-1323 | 67 |
| hsa-miR-24 | 735 | hsa-miR-483-3p | 67 |
| hsa-miR-10a | 716 | hsa-miR-17* | 63 |
| hsa-miR-30d | 705 | hsa-miR-99a | 62 |
| hsa-miR-99b | 646 | hsa-miR-17 | 61 |
| hsa-miR-215 | 631 | hsa-miR-1291 | 60 |
| hsa-miR-143 | 606 | hsa-miR-548c-5p | 59 |
| hsa-miR-19b | 601 | hsa-miR-548t | 59 |
| hsa-miR-30e | 588 | hsa-miR-1255b | 57 |
| hsa-miR-193b* | 561 | hsa-miR-146a | 56 |
| hsa-miR-23a | 541 | hsa-miR-30b* | 54 |
| hsa-miR-302a* | 533 | hsa-miR-28-3p | 53 |
| hsa-miR-27b | 530 | hsa-miR-3154 | 52 |
| hsa-miR-10b | 528 | hsa-miR-361-5p | 52 |
| hsa-miR-320d | 504 | hsa-miR-1285 | 52 |
| hsa-miR-27a | 492 | hsa-miR-22* | 49 |
| hsa-miR-25 | 490 | hsa-miR-126* | 48 |
| hsa-miR-93 | 442 | hsa-miR-3613-3p | 48 |
| hsa-miR-744 | 440 | hsa-miR-574-5p | 48 |
| hsa-miR-423-3p | 439 | hsa-miR-155 | 45 |
| hsa-miR-424 | 430 | hsa-miR-330-3p | 41 |
| hsa-miR-106b | 383 | hsa-miR-323b-3p | 39 |
| hsa-miR-148a | 358 | hsa-miR-181b | 38 |
| hsa-miR-206 | 322 | hsa-miR-885-3p | 32 |
| hsa-miR-30a | 293 | hsa-miR-144* | 32 |
| hsa-miR-222 | 284 | hsa-miR-3928 | 31 |
| hsa-miR-372 | 259 | hsa-miR-1250 | 28 |
| hsa-miR-181a | 254 | hsa-miR-151-5p | 22 |
| hsa-miR-125a-5p | 253 | hsa-miR-877 | 22 |
| hsa-miR-200b | 240 | hsa-miR-99b* | 16 |
| hsa-miR-92b | 230 | hsa-miR-148b | 14 |
| hsa-miR-144 | 220 | hsa-miR-409-3p | 12 |
| hsa-miR-26a | 220 | hsa-miR-3690 | 11 |
| hsa-miR-98 | 217 | hsa-miR-628-3p | 10 |
| hsa-miR-128 | 214 | hsa-miR-127-3p | 7 |
| hsa-miR-499-5p | 211 | hsa-miR-20b* | 6 |
| hsa-miR-183 | 197 | hsa-miR-302c | 5 |
| hsa-miR-503 | 182 | hsa-miR-92a-1* | 4 |
| hsa-miR-378 | 181 | hsa-miR-1224-5p | 4 |
| hsa-let-7d* | 170 | hsa-miR-3615 | 3 |
| hsa-miR-26b | 169 | hsa-miR-302a | 3 |
| hsa-miR-92b* | 169 | hsa-miR-432 | 3 |
| hsa-miR-1246 | 165 | hsa-miR-9 | 3 |
| hsa-miR-199b-5p | 161 | hsa-miR-184 | 2 |
| hsa-miR-182 | 160 | hsa-miR-323-3p | 2 |
| hsa-miR-889 | 156 | hsa-miR-516b | 2 |
| hsa-miR-151-3p | 155 | hsa-miR-493* | 2 |
| hsa-miR-106b* | 154 | hsa-miR-668 | 2 |
| hsa-miR-139-3p | 148 |  |  |
